# The sensitivity and stability of bacterioplankton community structure to wind-wave turbulence in a large, shallow, eutrophic lake

**DOI:** 10.1038/s41598-017-17242-z

**Published:** 2017-12-04

**Authors:** Jian Zhou, Boqiang Qin, Xiaoxia Han, Decai Jin, Zhiping Wang

**Affiliations:** 1 Taihu Laboratory for Lake Ecosystem Research, State Key Laboratory of Lake Science and Environment, Nanjing Institute of Geography and Limnology, Chinese Academy of Sciences, 73 East Beijing Road, Nanjing, 210008 China; 2 Key Laboratory of Environmental Biotechnology, Research Center for Eco-Environmental Sciences, Chinese Academy of Sciences, Beijing, 100085 China; 3 School of Environmental Science and Engineering, Shanghai Jiao Tong University, Shanghai, 200240 China

## Abstract

Lakes are strongly influenced by wind-driven wave turbulence. The direct physical effects of turbulence on bacterioplankton community structure however, have not yet been addressed and remains poorly understood. To examine the stability of bacterioplankton communities under turbulent conditions, we simulated conditions in the field to evaluate the responses of the bacterioplankton community to physical forcing in Lake Taihu, using high-throughput sequencing and flow cytometry. A total of 4,520,231 high quality sequence reads and 74,842 OTUs were obtained in all samples with *α*-*proteobacteria*, *γ*-*proteobacteria* and *Actinobacteria* being the most dominant taxa. The diversity and structure of bacterioplankton communities varied during the experiment, but were highly similar based on the same time of sampling, suggesting that bacterioplankton communities are insensitive to wind wave turbulence in the lake. This stability could be associated with the traits associated with bacteria. In particular, turbulence favored the growth of bacterioplankton, which enhanced biogeochemical cycling of nutrients in the lake. This study provides a better understanding of bacterioplankton communities in lake ecosystems exposed to natural mixing/disturbances.

## Introduction

Different microbiological studies aiming at biomedical, environmental, agricultural, and bioenergy applications share a common challenge: predict how microbial community functions and composition respond to disturbances^1^. Microbial communities are at the center of all ecosystem functions, and their responses to disturbances influence ecosystem stability and recovery^2^. In particular, since each bacterial taxa have distinct metabolic capabilities, alterations to the community would then yield changes in the range of functions that they could also perform^3–6^. Previous studies have investigated the relationships between various environment parameters and bacterial community composition (BCC) in various aquatic ecosystems and experimental systems. It has been shown that abiotic as well as biotic factors influence temporal and spatial shifts in microbial communities in aquatic environments^7^, and would therefore have potential implications to ecosystem functions^8^. As a consequence, microbial ecologists have been looking at the mechanisms that drive bacterial community assembly and how they affect the function and ecology of aquatic ecosystems^7^.

It has long been acknowledged that “there is no life without water, and there is no life in water without turbulence”^9^. Turbulence is an intrinsic and ubiquitous feature of bacterioplanktonic habitats^10^. Wind-driven turbulence and the associated environmental conditions of a mixed water column are important disturbances for bacterioplankton communities, such as in lakes^11,12^. Wind-wave turbulence directly impacts migration abilities, spatial distribution, and fundamental processes including motility, chemotaxis, and nutrient uptake of bacterioplankton^12^. Turbulence also drives bacterial distribution, affecting a range of bacterial processes, including resource competition, encounter rates with viruses, predators and conspecifics, and chemical signaling including quorum sensing and allelopathy^10^. Moreover, it may also directly or indirectly affect bacterial dynamics, activity and mortality^13,14^, and their abundance, growth and respiration by altering food web interactions^15,16^. Malits, *et al*.^14^ showed that small-scale turbulences could directly increase bacterial cell size and alter their morphology. From an ecological perspective, understanding the responses of bacterioplankton towards wind-induced turbulence could also provide unique insights into the underlying mechanisms of disturbances. Thus, turbulence in lakes and their microbial communities together could be an ideal model system to investigate disturbance ecology^17^. However, despite the widespread occurrence and implications of wind wave turbulence, its physical effects on microorganisms are rarely considered in microbial studies. It was only in the past few years that this theme has really come to the forefront^10^.

Climate change, which has repercussions on the world’s atmospheric system and weather patterns, may change the hydrodynamics of aquatic systems by increasing the frequency and intensity of tropical cyclones^18,19^. These physical disturbances strongly affect the stability of the water column and turbulent conditions in lakes. Because the fetch length of wind is long, large shallow lakes are strongly influenced by wind waves, which in turn are significant in the stability and evolution of lake eco-environment^20^. In recent years, eutrophication and consequential algal blooms in aquatic systems have become a major environmental issue that altered ecosystems and threatened human lives^21^. During these events, bacterioplankton are closely linked to algal bloom as they play key roles in major metabolic activities in the lake (i.e. nutrient cycles). Therefore, bacterioplankton response to wind waves is also important to understand emergent properties of the lakes ‘microbial loop’^6,22,23^. For example, in large and shallow lakes, changes in the physico-chemical characteristics of the water column due to wind waves^24,25^ alter the rates of bacterial activity and nutrient flow through benthic/planktonic microbial communities^26^, enhancing the decomposition rates of organic matter mediated by microbial enzymes^27^. We wondered how do the changes of BCC and function affect the matter degradation and nutrient cycling, and ultimately affect the growth of phytoplankton (especially harmful cyanobacteria). Indeed, recent works suggest that bacterioplankton, particularly the heterotrophic groups, may be essential for cyanobacteria biomass growth^28^. This highlights the importance of understanding the ecology of bacterioplankton community.

To address these knowledge gaps, we investigated the changes in bacterial community composition and their abundances under wind-driven wave turbulent conditions in a large, shallow, and eutrophic lake concordant with cyanobacterial bloom periods. To do this, we used high-throughput Illumina MiSeq sequencing and flow cytometry to generate profiles of the BCC before, during, and after exposure to turbulence. This approach has enabled us to examine not only the growth of bacterioplankton, but also their community composition and potential roles in driving algal blooms under wind wave environments. Understanding the effects of turbulence on bacterial community across time and space will provide further insights into the ecology of microorganisms, as well as enhance the ability to predict microbial responses to environmental changes, especially in the wake of the changing climate^8^.

## Materials and Methods

### Experimental design

Lake Taihu (Taihu), China’s third largest freshwater lake, is a shallow eutrophic lake with an average depth of 1.9 m, a maximum depth of 2.6 m and a total surface area of 2,230 km^2^, which is strongly influenced by wind-driven wave turbulence^29^. It is a major source of drinking water, livelihood, and food supply of more than 8 million people from the surrounding communities^30^. However, in the last decade, Taihu has experienced *Microcystis* bloom events on an unprecedented scale. In 2007 authorities shut down channels for water intake, resulting to a freshwater shortage for the 4 million residents of the city of Wuxi^31^.

To investigate changes in bacterial communities with disturbance, we used mesocosm set-ups in the Taihu Laboratory for Lake Ecosystem Research (TLLER), located at the shore side of Meiliang Bay (31°41′835″N, 120°22′044″E). The experiment was carried out in 12 acid-cleaned transparent glass mesocosms with 60 × 30 × 70 cm dimensions (Fig. S1, detailed in our previous studies^32,33^) from July 7^th^ to July 16^th^ 2014. Surface lake water (0.2 m, ~96 L) was pumped directly into each mesocosm from the nearshore (10 m away) of Meiliang Bay, Lake Taihu. Submerged wave-maker pumps (WP, Jiebao, China; Fig. S1C) were fixed under the water surface with magnets in each mesocosm to create hydrodynamic turbulence similar to wind-induced waves^32–34^. The mesocosms were floated and fixed in an outside artificial pond (10 × 10 × 2 m) which was filled with lake water, to mimic natural field conditions.

In shallow lakes, because turbulence has little space in which to dissipate, the turbulent kinetic-energy content is rather high^20^. The different levels of turbulence intensities used in this study were based on actual conditions previously observed in Lake Taihu in summer 2013. Turbulence intensity was determined using an acoustic Doppler velocimeter (10 MHz ADVField, Sontek/YSI, San Diego, California, USA), following the methods fully detailed in our previous studies^32,33^ (Supplementary materials and methods). In Taihu, the corresponding energy dissipation rates (*ɛ*) significantly varied from 6.01 × 10^−8^ to 2.39 × 10^−4^ m^2^ s^−3^ (Table 1), which corresponded to the range of values (from 1.07 × 10^−7^ to 6.67 × 10^−3^ m^2^ s^−3^) previously measured in the large, shallow Lake Balaton in Hungary^20^. Based on the *ɛ* values in Taihu, the *ɛ* used in this study (treatments) were 1.12 × 10^−6^ (low), 2.95 × 10^−5^ (medium), and 1.48 × 10^−4^ m^2^ s^−3^ (high), respectively (Table 1). These *ɛ* had corresponding Reynolds (*Re*) values that ranged from 5500 to 92620 (Table 1). The set-up without any hydrodynamic turbulence served as the control treatment (calm). All treatments were conducted in triplicate.

**Table 1:** Summary of the energy dissipation rates (*ɛ*) and Reynolds numbers (*Re*) of the four levels of turbulence (calm water, low, medium and high turbulent kinetic energy) used in the experiments and in Lake Taihu.

Turbulence level	*ɛ* (m^2^ s^−3^)	*Re*
Calm	0	0
Low	1.12 × 10^−6^	5,500
Medium	2.95 × 10^−5^	16,371
High	1.48 × 10^−4^	92,620
Lake Taihu	6.01 × 10^−8^–2.39 × 10^−4^	2,882–180,941

Water samples were collected daily by collecting 0.75 liter of vertically integrated water using a tube sampler between 7:00 and 8:00 in the morning, and immediately brought to laboratory for further processing and analysis. All sample containers, pipettes, test tubes and quartz micro-cuvettes were always rinsed with 10% hydrochloric acid and deionised water to prevent contaminations.

### Physico-chemical analysis

Water temperature (WT), dissolved oxygen (DO) and pH were measured with 6600 multi-sensor sonde (Yellow Springs Instruments, San Diego, California, USA). Nutrients including total nitrogen (TN), total dissolved nitrogen (TDN), ammonium (NH_4_^+^-N), nitrate (NO_3_^−^-N), nitrite (NO_2_^−^-N), total phosphorus (TP), total dissolved phosphorus (TDP), soluble reactive phosphorus (SRP), dissolved organic carbon (DOC) and chlorophyll *a* (Chl *a*), were analyzed following the methods described in Zhu *et al*.^35^. The particulate fractions of nitrogen (PN) and phosphate (PP) were obtained by subtracting the TDN/P from the TN/P.

### The abundance of bacterioplankton and active bacterioplankton

For the bacterial cell counts, 1 ml of water samples were fixed with filter-sterilized formaldehyde at a final concentration of 4%, and stored overnight at 4 °C, and subsequently frozen at −80 °C. Then, 500 μl of the sample was diluted 10-fold with ultrasonic processing (separate bacteria) for 10 min, and then filtered through a 48 µm mesh net. 1 ml of the filtrate was stained with SYBR^®^ Green I and then incubated in the dark for at least 15 min at room temperature before counting. The samples were analyzed on a FACSC JAZZ flow cytometry (BD Bioscience, San Jose, CA, USA) using FACS software (BD Bioscience), detailed in Gong, *et al*.^36^.

Active bacterioplankton were characterized by their capability to of assimilate and reduce the redox dye 5-cyano-2,3-ditolyl tetrazolium chloride (CTC, Sigma-Aldrich) manifesting as red-fluorescent deposits inside the cells, which were easy to detect and quantify by flow cytometry^37,38^. Briefly, each sample was incubated with 5 mM CTC (final concentration) at room temperature for 3 h in the dark. The reaction was stopped by the addition of 0.2 µm filtered 4% formalin (final concentration) and stored overnight at 4 °C, and subsequently frozen at –80 °C. 500 μl of the sample was diluted 10-fold with ultrasonic processing (separate bacteria) for 10 min, and then filtered through a 48 µm mesh net. Finally, 1 ml of each sample was immediately analyzed on a FACSC JAZZ flow cytometry using FACS software^36^.

### DNA extraction, PCR and high-throughput sequencing

Around 100 to 150 mL of each sample was immediately filtered onto 0.2 µm polycarbonate membranes (47 mm diameter, Millipore, Billerica, MA, USA) and frozen at –80 °C until further processing. We used the mixed DNA from the triplicates collected from the same treatment. The total genomic DNA from the 37 samples was separately extracted using FastDNA^®^ Spin Kit for Soil (MP Biomedicals) following the manufacturer’s instructions.

The hypervariable V4 region (about 207 bp) of bacterial 16 S rRNA gene was amplified using the forward primer 5′ - AYTGGGYDTAAAGNG - 3′, and the reverse primer 5′- TACNVGGGTATCTAATCC - 3′^39^. Each DNA sample was separately PCR-amplified in triplicate 25 μl reactions containing 1 × PCR buffer, 2.5 mM dNTPs, 0.625 U of Taq DNA polymerase, 10 μM of each primer, and 20 ng of target DNA. The following PCR cycle was used: initial denaturation at 94 °C for 5 min, followed by 25 cycles of 30 s at 94 °C, 30 s at 50 °C and 30 s at 72 °C. After the PCR, the amplicons were subjected to a final 7 min extension at 72 °C.

The total DNA and amplicons were sent to the Personal Biotechnology Co., Ltd. in Shanghai, China for high-throughput sequencing in an Illumina MiSeq (San Diego, CA, USA) platform using a paired-end 150-bp sequence read run. The sequence data generated in this study were deposited in NCBI under the project number SRR6237140-SRR6237176.

### Sequence analysis

Quality filtering of reads were performed in Quantitative Insights in Microbial Ecology^40^. Sequences were grouped into operational taxonomic units (OTUs) using UCLUST at a 97% similarity cutoff^41^. OTU-based community diversity indices (Chao1 estimator and Shannon index) and rarefaction curves of each sample were generated using the MOTHUR program^42^. A representative sequence for each OTU was selected, and the RDP classifier was used to assign taxonomic identity to each representative sequence^43^.

All statistical analyses were performed in R version 3.4.0^44^. Analysis of variance (ANOVA) was performed to test whether the environment parameters and bacteria abundance varied significantly among different treatments. Correlations between bacterioplankton and environment parameters were examined by Spearman’s rank correlation analysis. Principal coordinates analysis (PCoA) was performed with Bray Curtis dissimilarity distances performed in ‘vegan’ package in R^45^. Two-way crossed analysis of similarity (ANOSIM) was used to compare the similarity of the bacterial community between each treatment. Heat map of the most 50 abundant bacterial genus was constructed using “heatmap3” package also in R^46^.

## Results

### Dynamics of environmental parameters and bacterial abundance

Most of the environmental parameters varied during the experiments (Table S1). Water temperature generally ranged from 25.6 to 30.2 °C (ANOVA, *p* > 0.05). DO and pH were higher in the calm set-up than in the treatments (low, medium, and high turbulence) during the experiment. The concentration of TN and TP ranged from 1.5 to 2.0 mg L^−1^ and from 33.3 to 91.5 μg L^−1^, respectively. Also, the average concentrations of different nitrogen (TN, PN, TDN, NH_4_^+^-N, NO_3_^−^-N, and NO_2_^−^-N) and phosphorus (TP, PP, TDP, and SRP) species varied among treatments. However, these parameters remained similar, with only DO and some nitrogen showed significant differences among treatments (ANOVA, *p* < 0.05). Moreover, the 9-day average of pigment concentrations in the calm was lower compared to the treatments, and highest in medium (ANOVA, *p* < 0.05).

**Figure 1:**
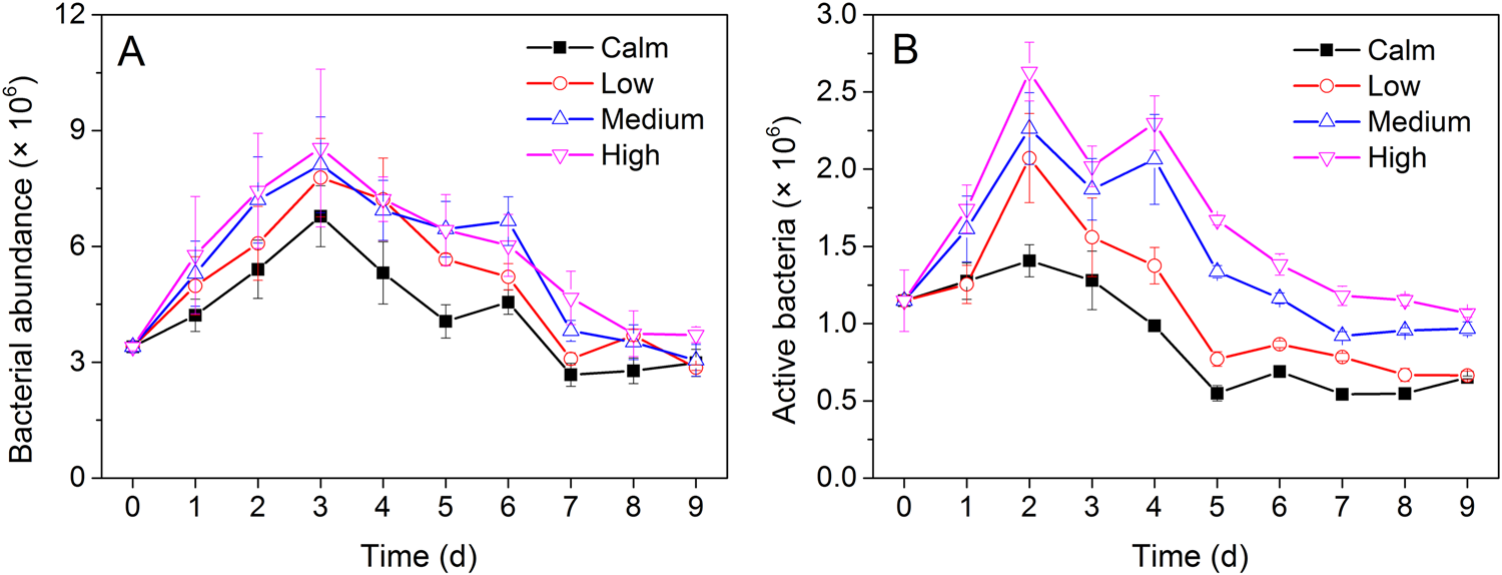
The abundance variation of total bacteria (**A**) and active bacteria (**B**) in the calm and turbulent treatments (low, medium, and high) during the experiments.

The abundance of total and active bacteria in all treatments were first increased before 2 or 3 days and then decreased, which were both higher in the turbulent treatments than in calm (Fig. 1). The 9-day average of total bacteria in calm, low, medium, and high treatments were 4.2 × 10^6^, 5.0 × 10^6^, 5.4 × 10^6^, and 5.7 × 10^6^ cells mL^−1^, with no significant difference between each treatments (ANOVA, *p* > 0.05). Moreover, The 9-day average of active bacteria in calm, low, medium, and high treatments were 9.1 × 10^5^, 1.1 × 10^6^, 1.4 × 10^6^, and 1.6 × 10^6^ cells mL^−1^, but notably most significantly different between calm and high (ANOVA, *p* < 0.05). On average, the relative abundance of active bacteria ranged from 14.5% to 34.1% (ANOVA, *p* > 0.05). Spearman’s rank correlation analysis further showed that Chl *a* was positively correlated with both total (r = 0.765, *p* < 0.001) and active (r = 0.821, *p* < 0.001) bacteria.

#### Bacterial community structure

**Figure 2:**
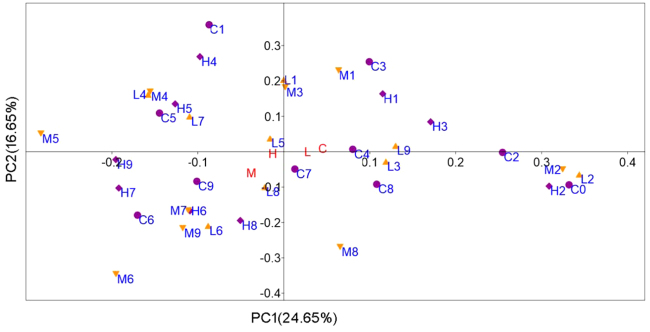
Principal co-ordinates analysis of bacterial community in the calm and three turbulent treatments during the experiments, where M5 is the sample of M, at day 5. C, L, M, and H represent the calm (circle), low (triangle), medium (inverted triangle), and high (diamond) turbulence treatments, respectively.

After processing, 4,520,231 high quality sequences remained with an average length of 225 bases. A total of 74,842 OTUs were generated after clustering at a 97% similarity level, and 4,414 OTUs were singletons. Rarefaction curves of all samples nearly approached a plateau, suggesting that these communities had nearly been well sampled (Fig. S2). Further, BCC (defined as the relative abundance of OTUs) were assessed in different turbulence treatments during the experiment (Fig. 2). PCoA plots revealed distinct clustering (before and after day 4), suggesting succession of bacterial communities (Fig. 2). Notably, bacterial communities clustered based on the time of sampling rather than turbulence treatments (Fig. 2). This was also supported by the pairwise ANOSIM comparisons showing the significant similarities in bacterial communities among treatments (*p* < 0.001).

### Taxonomic identification and variation

**Figure 3:**
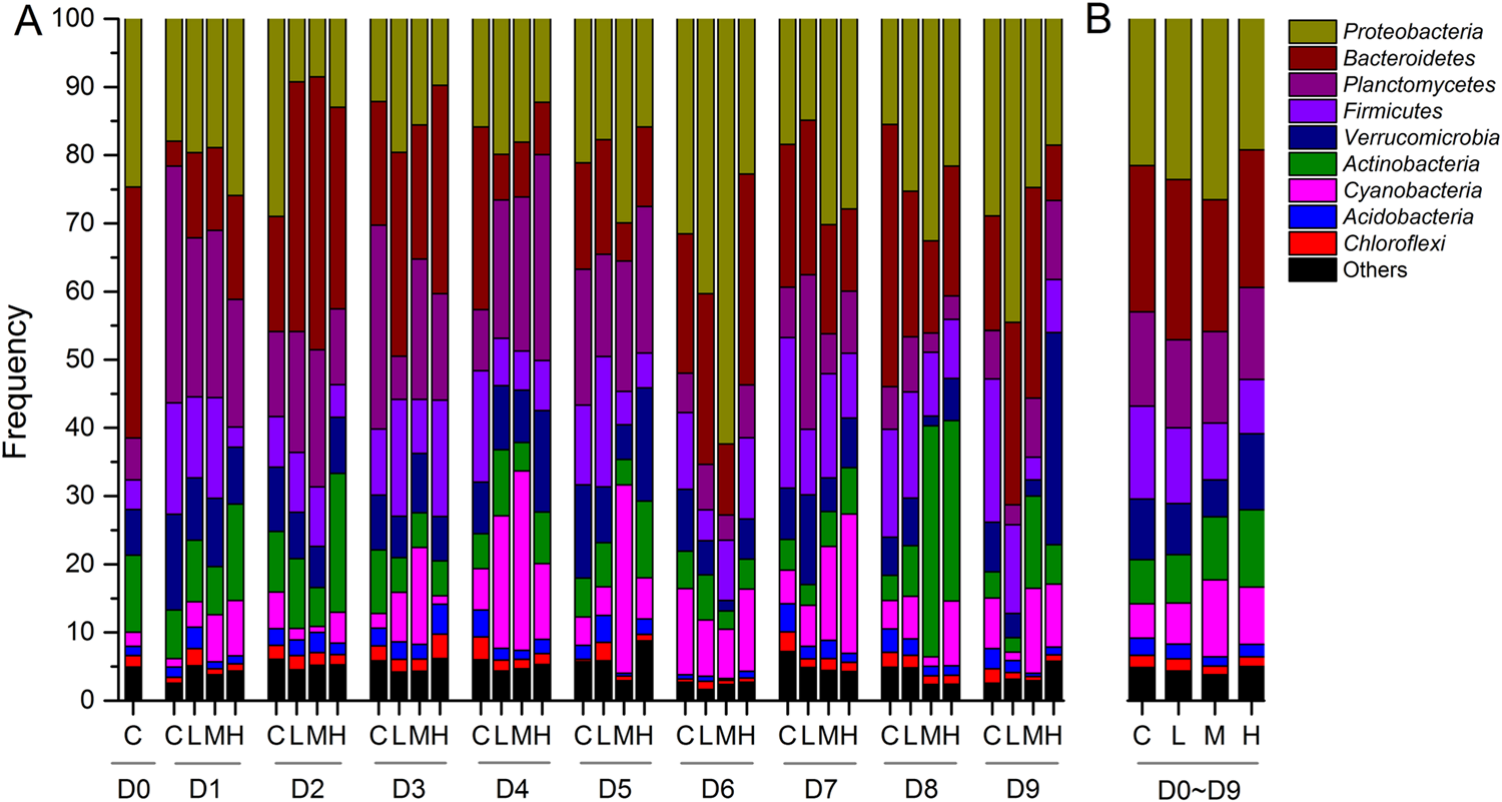
Bacterial community structure at the phylum level in the calm and three turbulent treatments separated according to time (day, (**A**)) and average percentage of the dominant taxa (**B**) during the experiments. Only the top 9 phylum are listed.

Overall, the OTUs were classified to belong to 60 phylum-level taxonomic groups based on all samples. The 9 most abundant bacterial phyla were *Proteobacteria*, *Bacteroidetes*, *Planctomycetes*, *Firmicutes*, *Verrucomicrobia*, *Actinobacteria*, *Cyanobacteria*, *Acidobacteria*, and *Chloroflexi* (Fig. 3). *Proteobacteria* (22.6%) dominated the community, 40.1% of which belonged to *α*-*proteobacteria* and 31.2% from *γ*-*proteobacteria*. Initially, the BCC (relative biomass expressed in %) mainly consisted of *Bacteroidetes* (36.8%), *Proteobacteria* (24.7%), and *Acidobacteria* (11.3%, Fig. 3A). During the study, the frequency of dominant phyla notably varied among the treatments (Fig. 3A). For example, the *Proteobacteria* was observed to increase from day 2 to 6 but returned to its the initial state towards the end of the study (Fig. 3A). Interestingly, the BCC in the different treatments displayed similar composition at the phylum levels during the experiments (Fig. 3B). Kruskal-Wallis rank test further revealed that there were no significant differences in the main 9 bacterial phyla among the treatments for the entire duration of the experiment (*p* > 0.05).

**Figure 4:**
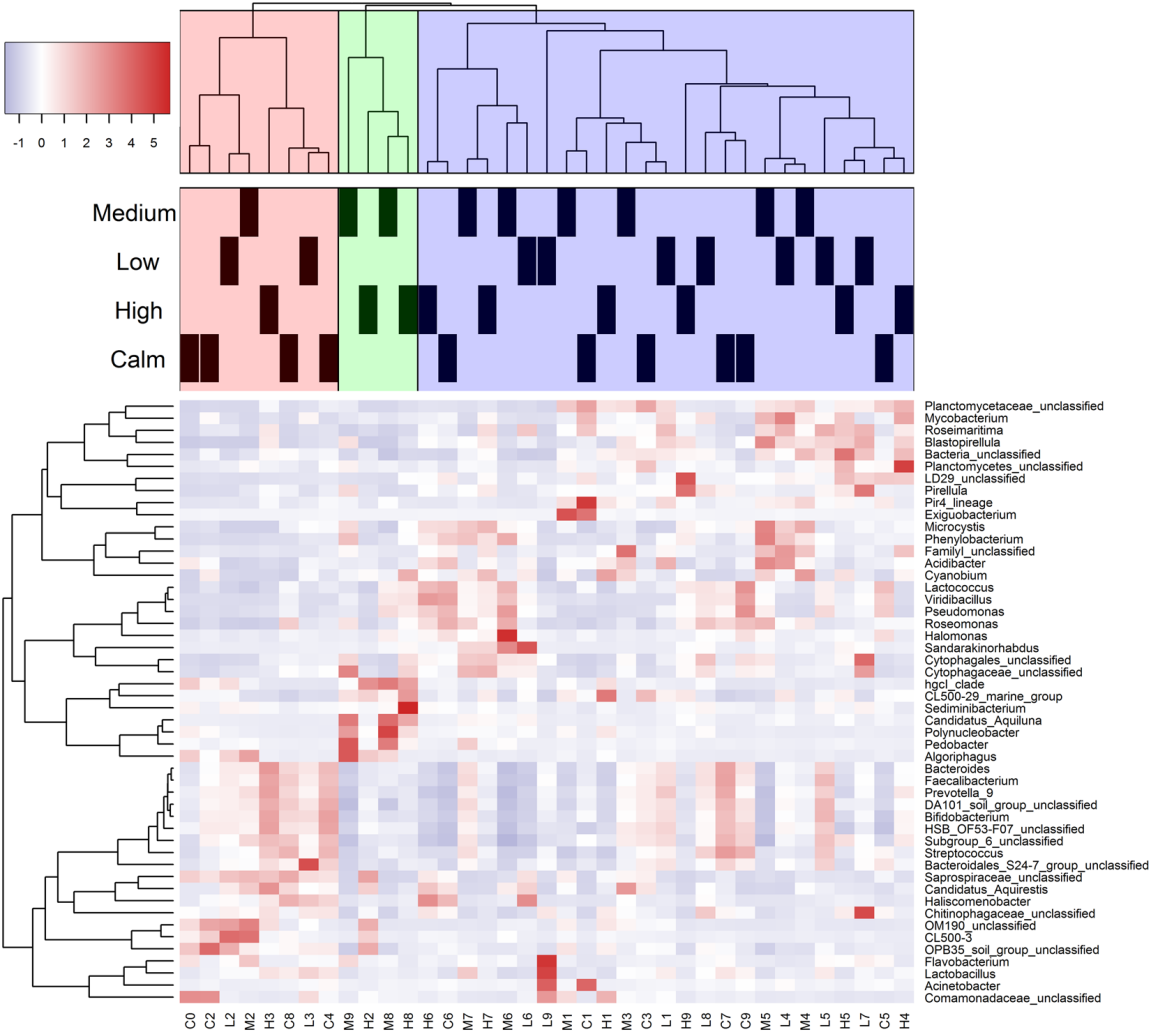
A heat map revealing the dynamics of the 50 most abundant bacterial genera (accounted for 64.5%) from the calm and three turbulence treatments during the experiments, where C2 is the sample of C, at day 2. C, L, M, and H represent the calm, low, medium, and high turbulence treatments, respectively.

At the genus level, the 50 most abundant genera comprised on average of 64.5% of the total OTU richness (Fig. 4), mainly associated with known freshwater lineages or clades, such as *Microcystis* and *Haliscomenobacter* ^47^. Within the *Actinobacteria*, the two most dominant genera were the hgcl_clade (28.8%) and *Bifidobacterium* (11.7%). The most abundant *Proteobacteria* were *Sandarakinorhabdus* (10.3%), *Phenylobacterium* (10.2%), and *Halomonas* (7.6%) and an unclassified genus (7.8%) belonging to family *Comamonadaceae*. All of the 50 dominant genera/lineages were present in all samples (*n* = 37) but only 3 (*Streptococcus*, Bacterioidales_S24–7_group, and *Pedobacter*) of the 50 most abundant genera were identified to be significantly different among treatments (ANOVA, *p* < 0.05).

The heat map showed the temporal differences in community structure among treatments of the most abundant taxa. These differences immediately manifested after day 0, followed by changes in patterns and clustering on the succeeding days with three levels of clustering in all samples (Fig. 4). Interestingly, BCC mainly remained similar among treatments but drastically changed and diversified after day 4 (Fig. 4), suggesting that the community structure of bacterioplankton shifted after 4 days. Specifically, a distinct cluster representing the samples collected after day 7 was observed in medium and high treatments (Fig. 4).

#### Bacterial community diversity

**Figure 5:**
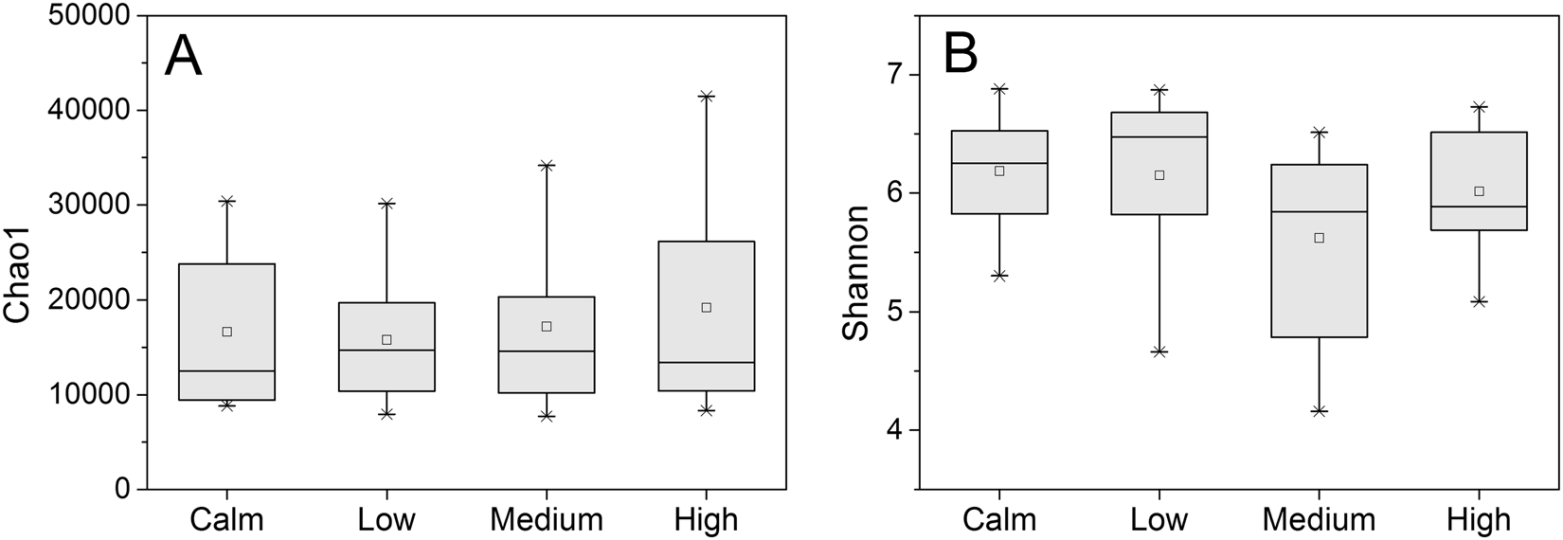
Bacterial community diversity (Chao1, (**A)**; Shannon, (**B**)) in the calm and three turbulent treatments (low, medium, and high) during the experiments.

During the experiments, diversity indices Chao1 and Shannon index ranged from 7724.9 to 41476.2 and from 4.2 to 6.9, but only with little variation among the treatments (Fig. 5, *p* > 0.05). This also suggests that hydrodynamic turbulence have no effect on bacterial community diversity.

## Discussion

Hydrodynamic processes are an important feature of the ecology of microorganisms and can have far-reaching and hitherto, largely unrecognized consequences in natural ecosystems such as oceans and lakes^10^. In the wake of the changing climate, wind conditions are predicted to be altered significantly, which in turn will lead to changes in the wind wave turbulence that the plankton communities experience^48^. However, most of the studies on bacterial community structure were based on field investigations, where interpretations were mostly inferential. The direct effects of natural turbulences on heterotrophic bacterial communities remain controversial^14,49,50^. Disentangling the ecological effects of wind wave turbulence can provide new insights on bacterioplankton responses to physical forcing in lakes, which may be essential for quantifying and understanding lake ecosystem process and its recovery from eutrophication. The usefulness of mesocosms as a tool to investigate community responses has been thoroughly tested, and the current gradient-based design is an effective tool for inferential studies of ecological processes^48,51^.

In this study, a turbulence gradient was introduced to natural plankton assemblages in unfertilized lake water in mesocosms to assess the effects of wind wave turbulence on the community structure of bacterioplankton in Lake Taihu. Results of our experiments showed that although the bacterioplankton composition significantly varied during the experiment, wind wave turbulence did not shift the competition among bacterioplankton species (Figs 2–4), suggesting insensitivity. Shade, *et al*.^52,53^ found that bacterial community composition were resistant to lake mixing even after strong mixing events such as typhoons. Also, although wind-driven turbulence was stronger in the central lake in Taihu, bacterioplankton community exhibited strong spatial similarity in the entire lake^17,54–56^.

In aquatic ecosystems, bacterioplankton communities primarily depend on changes occurring in both time and space^57,58^. However, responses of microbial communities to such changes are also mediated by their community characteristics, including their history, metabolic flexibility, physiological tolerance, dispersal capacity, and taxonomic and functional diversity^59–61^. Generally, this study suggests that bacterioplankton communities are not sensitive to wind wave turbulence in the lake, which could be associated with their high abundance, widespread dispersal, potential for rapid growth, and rapid evolutionary adaptation through mutations or horizontal gene transfer. These characteristics allow bacteria to quickly adapt to new environmental conditions and maintain the stability of community^57–59,62^. For example, due to their small bodies, free-living bacteria are often assumed to be ubiquitous, and they are presumably more likely to become widely dispersed^63^. Alternatively, the dispersal probability of bacteria is quite fast, which has important implications on the temporal dynamics of microorganisms^60^. Soininen^64^ highlighted factors, such as dispersal rate, as likely drivers of bacterial community turnover and variation in both space and time. High dispersal ability provides higher probability of colonizing suitable habitats from regional pools, thereby potentially reducing the variation of community composition through time^65,66^. Recently, Tang, *et al*.^56^ investigated the bacterial spatiotemporal dynamics in Taihu, and found that community composition showed strong similarity in the different hydrodynamic regions of the lake. Nelson, *et al*.^67^ illustrated the contrasting roles of environmental selection and dispersal in structuring bacterioplankton communities, supporting a weaker effect of advective flow on bacterioplankton communities among lakes. Therefore, bacterial communities may be less sensitive to wind wave turbulence, allowing the bacterial communities to vary in abundance but not in composition even under turbulent conditions.

Although traditionally, the small size and physiology of microscopic organisms is not affected by the moving fluid at the micro-scale, turbulence significantly triggered higher bacterial abundance in the turbulent treatments in the present study (Fig. 1). This is consistent with results of previous studies showing that small-scale turbulence has a positive effect on bacterial abundance^10,15,68,69^. One suggested explanation is that turbulence changes the encounter rates between heterotrophic microplankton and their more preferred prey (picoplankton and nanoflagellates), thus releasing bacteria from grazing pressure under turbulent conditions^15^. Moreover, turbulence affects competition for resources^14^, even though it is generally considered that bacterial uptake is inconsequential since their size is considerably smaller than the Kolmogorov scale. Also, turbulence may indirectly enhance the growth of bacteria. Altering top-down control, based on grazing shift hypothesis, turbulence may change nutrient conditions. For instance, if turbulence favors the growth of large-celled phytoplankton which excrete high-molecular weight dissolved organic matter^70,71^, bacteria may in fact benefit from the increased flux of organic matter under turbulent conditions. Therefore, microorganisms exposed to turbulent flows enhance their growth and activity by being released from grazing pressure, increasing nutrient uptake, and changing the nutrient conditions of their environment. Moreover, in the strong turbulent treatments (medium and high), the sedimentary bacteria resuspended through mixing were partly contributed to the high bacterial abundance, as seen from the presence of sediment-associated bacteria (*Sediminibacterium* and *Pedobacter*) that dominated in medium and high turbulent conditions at the end of the experiments.

In lakes, interactions between phytoplankton and bacteria have been proposed to also influence bacterial community dynamics^72–76^. Although diatoms and green algae tend to be superior under mixed or turbulent conditions^48,77,78^, gas-vacuolated *Microcystis* has been mainly promoted by wind wave turbulence, which also dominated in the turbulent conditions before about 8 days during the experiment^79^. Under turbulent conditions, bacterioplankton community was observed to be more consistent than the phytoplankton and zooplankton community^79,80^, indicating differences in stability or stochasticity of plankton communities in response to environmental changes^62^. This also indicates that bacteria could be less sensitive to environmental changes than the eukaryotic groups^62^. Moreover, regardless of nutrient conditions, Chl *a* and bacterial production rates were positively correlated with turbulence, where bacterial abundance and activity were higher under turbulent conditions. This suggests that turbulence may accelerate nutrient cycling, and may play an important role in nutrient strategies of plankton.

In conclusions, changes in bacterial production occurred under turbulent conditions without changes in community structure and diversity in Lake Taihu during cyanobacterial bloom periods. Wind wave turbulence favored the accumulation of bacterioplankton especially active bacterioplankton, which benefited the biogeochemical cycling of nutrients in the lake. However, turbulence did not shift the competition among bacterioplankton species. The direction and variation of bacterioplankton community dynamics were main accordance under different turbulent conditions. Moreover, compared to the phyto- and zooplankton, bacteria were less-sensitive indicators of the prevailing turbulent conditions at the community level in Lake Taihu. This body of work represents only a snapshot of the range of microbial processes that are influenced by fluidic mechanical forces and transport. Bacterial community response to lake mixing is additionally relevant for understanding microbial ecology of disturbance and stability.

## Electronic supplementary material

Supporting Information
