# Supplementary material for: The sensitivity and stability of bacterioplankton community structure to wind-wave turbulence in a large, shallow, eutrophic lake
Source: Sci Rep. 2017 Dec 4;7:16850. doi: 10.1038/s41598-017-17242-z (PMC5715125; doi:10.1038/s41598-017-17242-z)
Supplement: Supplementary file 1 — Supporting Information [file 41598_2017_17242_MOESM1_ESM.pdf]

Supporting Information

The sensitivity and stability of bacterioplankton community structure to wind-wave turbulence in a large, shallow, eutrophic lake

Jian Zhou[1], Boqiang Qin[1*], Xiaoxia Han[1], Decai Jin[2], Zhiping Wang[3]

[1]Taihu Laboratory for Lake Ecosystem Research, State Key Laboratory of Lake Science and Environment, Nanjing Institute of Geography and Limnology, Chinese Academy of Sciences, 73 East Beijing Road, Nanjing 210008, China.

[2]Key Laboratory of Environmental Biotechnology, Research Center for Eco-Environmental Sciences, Chinese Academy of Sciences, Beijing 100085, China.

[3]School of Environmental Science and Engineering, Shanghai Jiao Tong University, Shanghai 200240, China.

*Corresponding author: qinbq@niglas.ac.cn;

1. SUPPLEMENTARY MATERIALS AND METHODS

1.1 Experimental setup

Mesocosm experiments were carried out in as a total of twelve (12) customized tanks made of 8 mm thick Plexiglass that have maximal capacities of 126 L (Fig. S1). Frequency conversion wave-maker pumps (WP, Jebao, China; Fig. S1c) were used to generate artificial waves. Rebounds were reduced by the slopes (5:1) on the sides (Fig. S1a), where energy dissipation plates are also fixed (Fig. S1b). The tanks were immersed in water for 15 days before being used for any experiment to remove contaminants.

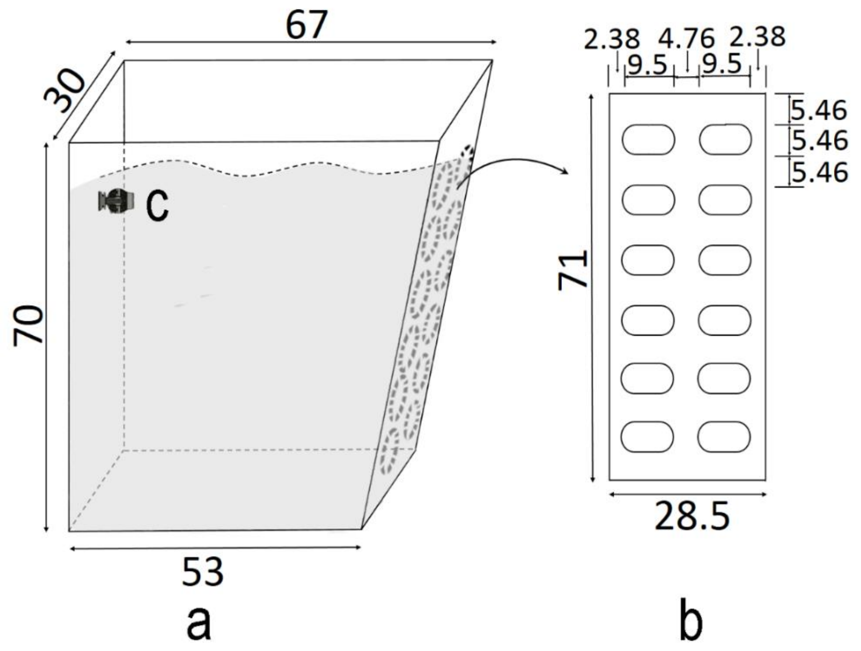

Figure S1 Diagram of the mesocosm tank (a) used in the experiments which includes an energy dissipation plate (b) and a wave-maker pump (c). All dimensions are in centimeter.

1.2 Turbulence measurements

The submerged wave-maker pumps fixed under surface water by strong magnets, were used to generate turbulence simulating the ones induced by natural wind waves as demonstrated in previous studies[1-4].

The pump frequency was set to 1 Hz and the turbulence generated was monitored and measured by an acoustic-Doppler velocimeter (ADV, 10 MHz ADVField; Sontek/YSI, San Diego, California, USA). Turbulent kinetic energy was measured from the middle of the tank

with a 25 Hz measurement for a period of 2 min. Measurements were performed after the turbulent motion in the tank had reached a steady state after around 10 min.

The root mean square velocity (U_{rms}), which helps define the characteristic speed of the turbulence, was calculated using the following formula:

$$U_{rms} = \sqrt{u_{rms}{}^2 + \mathrm{v}_{rms}{}^2 + \mathrm{w}_{rms}{}^2} \quad (1)$$

where

$$u_{rms} = \sqrt{\frac{\sum \mu_x{}^2 - (\sum \mu_x)^2/n}{n-1}}$$

$$v_{rms} = \sqrt{\frac{\sum \mu_y{}^2 - (\sum \mu_y)^2/n}{n-1}}$$

$$w_{rms} = \sqrt{\frac{\sum \mu_z{}^2 - (\sum \mu_z)^2/n}{n-1}} \quad (2)$$

is the fluctuation of the flow for Cartesian vector *x*, *y* and *z*, and *n* is the number of samples per measurement. The U_{rms} were expressed as averages for the whole tank. The energy dissipation rate (ε, $m^2\ s^{-3}$), which describes the rate at which the turbulent energy decays over time, was deduced from the U_{rms} ($m\ s^{-1}$) following the formula described by Sanford[5]:

$$\varepsilon = A_1 \frac{U_{rms}{}^3}{l} \quad (3)$$

where A_1 is an dimensional constant of order 1[6, 7], and *l* is the depth (m) of the water column describing the size of the largest vortices.

The Reynolds number (*Re*, the ratio of inertial forces to viscous forces) for the given turbulence levels was calculated following Peters and Redondo[8]:

$$Re = l \frac{U_{rms}}{v} \quad (4)$$

where *l* is the water depth (m) and *v* is the kinematic viscosity for water ($8.5 \times 10^{-7}\ m^2\ s^{-1}$).

Table S1 Mean and standard deviation of the physical, chemical and biological measurements with calm and three turbulence treatments (low, medium, and high) during the experiments. These include water temperature (WT), dissolved oxygen (DO), suspended solids (SS), total nitrogen (TN), total dissolved nitrogen (TDN), particulate nitrogen (PN), ammonium (NH_4^+-N), nitrate (NO_3^--N), nitrite (NO_2^--N), total phosphorus (TP), total dissolved phosphorus (TDP), particulate phosphorus (PP), soluble active phosphorus (SRP), dissolved organic carbon (DOC), chlorophyll *a* (Chl *a*).

	Parameter	Control	Low	Medium	High
Physical	WT (℃)	27.5 ±1.3	27.5 ±1.4	27.5 ±1.4	27.5 ±1.4
	DO (mg L^{-1})	8.6 ±1.3	7.7 ±0.4	7.8 ±0.5	7.7 ±0.4
	pH	8.8 ±0.3	8.7 ±0.3	8.6 ±0.3	8.5 ±0.2
	SS (mg/L)	21.2 ±7.6	28.3 ±10. 1	36.1 ±5.0	31.4 ±14.3
Chemical	TN (mg L^{-1})	1.64 ±0.19	1.70 ±0.15	**1.85 ±0.10**	**1.83 ±0.08**
	TDN (mg L^{-1})	1.15 ±0.17	1.05 ±0.19	1.07 ±0.19	1.17 ±0.12
	PN (mg L^{-1})	0.49 ±0.07	**0.65 ±0.06**	**0.77 ±0.11**	**0.66 ±0.10**
	NH_4^+-N (mg L^{-1})	0.18 ±0.07	0.23 ±0.06	**0.27 ±0.04**	**0.26 ±0.05**
	NO_3^--N (mg L^{-1})	0.53 ±0.11	0.47 ±0.16	0.40 ±0.20	0.52 ±0.11
	NO_2^--N (mg L^{-1})	0.03 ±0.002	0.03 ±0.005	**0.03 ±0.008**	0.03 ±0.004
	TP (μg L^{-1})	52.1 ±22.5	57.7 ±19.4	63.4 ±15.5	61.4 ±18.8
	TDP (μg L^{-1})	11.5 ±6.8	12.7 ±6.6	13.1 ±6.4	14.7 ±5.1
	PP (μg L^{-1})	40.6 ±21.2	45.1 ±17.	50.2 ±15.7	46.7 ±16.9
	SRP (μg L^{-1})	4.5 ±4.9	4.8 ±5.0	5.2 ±5.3	5.1 ±5.3
	DOC (mg L^{-1})	4.3 ±0.2	4.4 ±0.2	**4.8 ±0.6**	4.7 ±0.5
Biological	Chl *a* (μg L^{-1})	20.0 ±6.3	26.8 ±9.1	**31.8 ±9.1**	29.7 ±12.8

Bold values indicate that there is a significant difference between the calm and the turbulent treatments, which was determined by ANOVA.

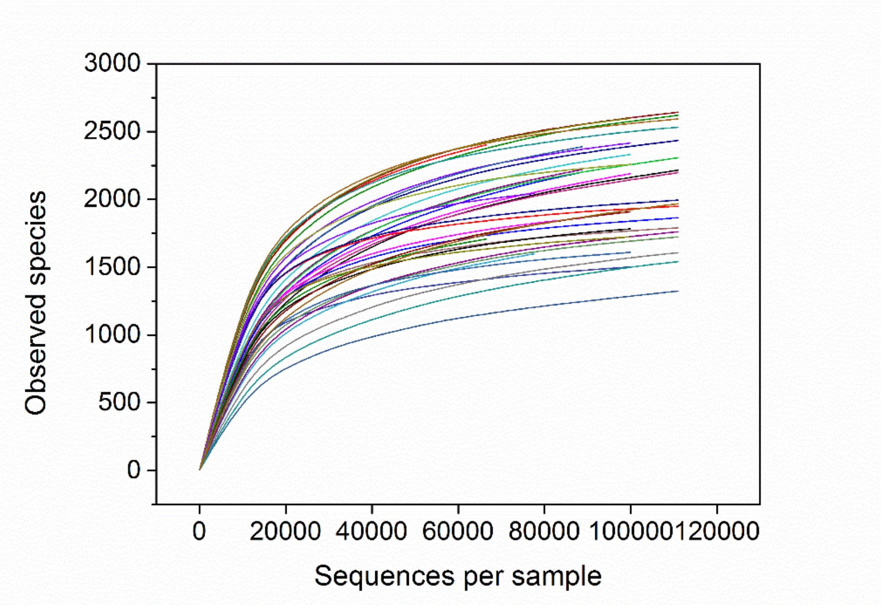

Figure S2 Rarefaction curves based on the number of OTUs observed for all samples in the calm and turbulent treatments during the experiment.

References

1. Pekcan-Hekim, Z., Joensuu, L., Horppila, J., Grant, J. Predation by a visual planktivore perch (*Perca fluviatilis*) in a turbulent and turbid environment. *Can J Fish Aquat Sci* **70**, 854–859 (2013).
2. Harkonen, L., Pekcan-Hekim, Z., Hellen, N., Ojala, A., Horppila, J. Combined effects of turbulence and different predation regimes on zooplankton in highly colored water—implications for environmental change in lakes. *PLoS ONE* **9**, e111942 (2014).
3. Zhou, J., Qin, B., Casenave, C., Han, X. Effects of turbulence on alkaline phosphatase activity of phytoplankton and bacterioplankton in Lake Taihu. *Hydrobiologia* **765**, 197–207 (2016).
4. Zhou, J. *et al*. Effects of wind wave turbulence on the phytoplankton community composition in large, shallow Lake Taihu. *Environ Sci Pollut Res* **22**, 12737–12746 (2015).
5. Sanford, L. P. Turbulent mixing in experimental ecosystem studies. *Mar Ecol Prog Ser* **161**, 265–293 (1997).
6. Moum, N. J. Energy-containing scales of turbulence in the ocean thermocline. *J Geophys Res* **101**, 14095–14109 (1996).
7. Kundu, P. K., Cohen, I. M. Fluid mechanics. San Diego Academic Press, San Diego (2010).
8. Peters, F., Redondo, J. M. Turbulence generation and measurement: application to studies on plankton. *Sci Mar* **61**, 205–228 (1997).
